# Supplementary material for: Exploratory study of using Magnetic resonance Prognostic Imaging markers for Radiotherapy In Cervix cancer (EMPIRIC): a prospective cohort study protocol
Source: BMJ Open. 2024 Apr 18;14(4):e077390. doi: 10.1136/bmjopen-2023-077390 (PMC11029356; doi:10.1136/bmjopen-2023-077390)
Supplement: Supplementary data [file bmjopen-2023-077390supp001.pdf]

Supplementary Table 1. EMPIRIC Study assessment schedule

Week		1					2					3					4					5					6
Procedure	Before the start of radiotherapy	Days 1-5					Days 8-12					Days15-19					Days 22-26					Days 29-33					Days 36-40
Consent & Eligibility assessment	✓																										
*Chemoradiotherapy (CRT)		✓	✓	✓	✓	✓	✓	✓	✓	✓	✓	✓	✓	✓	✓	✓	✓	✓	✓	✓	✓	✓	✓	✓	✓	✓	*Image-guided brachytherapy
MRI scanning: T1,T2, DCE,DWI,BOLD	✓										✓															✓	Routine MRI
Biopsy sample	From original diagnostic biopsy																										✓
Blood sample (CTC)	✓										✓															✓	

✓ Indicates when patient will receive stated treatment or undergo stated investigation

* Concurrent chemo-radiotherapy (EBRT plus image guided-brachytherapy with weekly cisplatin) is administrated per standard of care
